# A qualitative exploration of community stakeholders perspectives on dengue outbreak management in urban Nepal: navigational insights and challenges

**DOI:** 10.1186/s41182-025-00758-w

**Published:** 2025-05-30

**Authors:** Sushmita Ghimire, Devendra Raj Singh

**Affiliations:** 1 Department of Public Health, Asian College for Advance Studies, Lalitpur, Nepal; 2 Center for Health and Disease Studies-Nepal, Kathmandu, Nepal; 3 School of Human and Health Sciences, University of Huddersfield, Huddersfield, UK

**Keywords:** Dengue, Community members, Stakeholders engagement, Challenges, Experience, Nepal

## Abstract

**Introduction:**

Dengue outbreaks are a recurring public health challenge in urban areas of Nepal, necessitating proactive engagement of community stakeholders to ensure effective prevention and control measures. However, there is limited evidence of community engagement in dengue management in urban settings in Nepal. This study aimed to assess the community stakeholders’ perceptions and experiences on dengue outbreak management in urban settings in Nepal.

**Method:**

A qualitative study with interpretive phenomenology approach was conducted among community leaders and female community health volunteers (FCHVs) who were directly involved in the prevention and control of Dengue outbreaks in Lalitpur Metropolitan City. The participants were selected based on the purposive sampling with inclusion criteria. Twenty face-to-face, in-depth interviews were conducted between April 2023 and June 2023 among the local community stakeholders. The data were analysed using a thematic analysis approach guided by the Health Belief Model as a theoretical framework.

**Results:**

Findings are presented under two broader themes (i) Community contributions in awareness building, and (ii) Stakeholders’ experiences on dengue management and prevention. The study identified perceived severity and susceptibility, driven by recurrent dengue outbreaks, as key motivators for stakeholders engagement and actions in dengue outbreak management. Community-based initiatives, such as awareness programs and home-to-home visits, were considered effective in increasing public engagement. However, challenges such as delayed actions, the community’s limited knowledge of dengue prevention and control, reluctance for consistent source reduction, inadequate water supply, and adverse sociocultural practices posed significant barriers to dengue management. Despite these obstacles, stakeholders expressed strong self-efficacy and commitment to the prevention and control of potential dengue outbreaks in future.

**Conclusion:**

Local stakeholder engagement was considered crucial in dengue outbreak prevention and control. However, proactive, timely planning, continuous dissemination of dengue education, improved health infrastructures, and enhanced collaboration and coordination among community members and authorities are essential for the effective management of dengue outbreaks.

**Supplementary Information:**

The online version contains supplementary material available at 10.1186/s41182-025-00758-w.

## Introduction

The rapidly emerging mosquito-borne viral infection, dengue fever, poses a serious threat to public health globally, particularly in tropical and subtropical regions with poor health resources [1]. World Health Organization (WHO) estimates, 50–100 million cases of dengue fever are reported yearly, and over half of the world's population resides in dengue-endemic nations [2]. Most countries in Southeast Asia face a high burden of Dengue Fever (DF)/Dengue Hemorrhagic Fever (DHF) and experience frequent and cyclical epidemics [3]. If untreated, dengue can lead to severe, life-threatening conditions such as dengue hemorrhagic fever and dengue shock syndrome, although also imposing significant economic and social costs on affected communities [1].

Dengue was first detected in Nepal in 2004 [4–7], with cases increasing significantly over time. Outbreaks occur nearly every monsoon season, particularly in the Terai region and urban areas, with major outbreaks in 2007, 2009, 2010, 2013, 2016, 2019, 2020 and 2022 involving serotype DENV-1, DENV-2 and DENV-3 in several districts of Nepal [8–10]. The 2022 dengue outbreak in Nepal was the largest recorded since 2004, with 54,784 cases—sharply rising from 540 cases in 2021 and surpassing the previous peak of 17,992 in 2019 [10]. Due to factors like rising temperatures, growing urbanization, increased trade from afflicted regions, and inadequate health infrastructure, the Kathmandu Valley's metropolitan and municipality areas are particularly vulnerable to dengue outbreaks. These characteristics also create optimal conditions for the Aedes mosquito [11]. Although supportive and symptomatic treatment often work, the lack of specific antiviral or vaccine in Nepal results in increasing number of deaths yearly. Hence, the most effective strategies to combat the growing threat are environmental interventions, efficient mosquito control, and active community involvement in prevention and control efforts. Active community involvement and social participation play a vital role in preventing dengue [12, 13]. Effective dengue prevention and control require an integrated approach, including eliminating mosquito breeding sites, using insecticides and biological control, raising community awareness, promoting participation, and ensuring early diagnosis and treatment [14–17]. The community-based approaches through wider community engagement in the surveillance, source reduction, promoting preventive behaviours and facilitating the coordination of the response efforts during the outbreak is vital [15, 18]. Evidence from different studies in various countries demonstrated the importance of community engagement for the prevention and control of dengue fever [19–22]. Community stakeholders, including local leaders and health professionals, play a critical role in the prevention and management of dengue [23]. Coordination of community involvement in vector control initiatives, public awareness campaigns, and immediate response to outbreaks is necessary for effective dengue management [18]. Various studies showed that better adherence to preventative measures, prompt case reporting, and extensive outreach in high-risk areas are all ensured by community engagement [21, 23, 24]. However, the evidence of community engagement in dengue prevention and control has been lacking in the Nepalese context.

In the context of Nepal, Female Community Health Volunteers (FCHVs) have historically been effective in preventing and controlling various communicable diseases, such as diarrhoea, pneumonia, vector borne diseases and maternal and child health conditions, by delivering health education, promoting early health-seeking behaviour, and facilitating access to services at the grassroots level [25–27]. FCHVs play a critical role in dengue prevention and control at the community level by raising awareness, educating communities on preventive measures like eliminating mosquito breeding sites, and assisting in early detection efforts. Their demonstrated ability to enhance community health outcomes highlights their importance in dengue community management as well, assisting in bridging the gap between communities and health services, ensuring that prevention and control measures are implemented at the grassroots level [28]. However, their experiences and challenges faced in the prevention and control of dengue are not well explored and documented.

### Dengue prevention and control activities in Nepal

The Nepalese government emphasizes multilevel stakeholder coordination for effective dengue prevention and control, implementing initiatives such as community orientation, search and destroy campaigns, routine surveillance, data verification, and collaboration with schools and hotels for outbreak preparedness [6].

The"search and destroy"program is a key initiative implemented across all seven provinces to reduce dengue transmission by identifying and eliminating mosquito breeding sites. A budget has been allocated for 139 municipalities to support this effort [29]. Additionally, the Epidemiology and Disease Control Division (EDCD) provided funding to organize stakeholder meetings for advocacy on dengue prevention during the peak transmission months in the same municipalities, districts, and provinces [10]. In late August, large-scale search and destroy operations (prioritising main breeding containers) were launched in the Lalitpur and Kathmandu districts during a previous outbreak [10]. Additionally, virtual coordination between EDCD and local authorities ensures a unified response at both local and district levels to control the virus's spread [30].

Although community stakeholders play a crucial role in managing dengue epidemics, there is a noticeable lack of knowledge regarding their experiences and the difficulties they encounter. The medical and epidemiological facets of dengue have been the subject of several researches; however, the qualitative components of community engagement have received less attention. Learning from the experiences of community stakeholders can strengthen future interventions by offering valuable insights into the behavioural, social, and logistical challenges encountered during dengue outbreaks. A deeper understanding of the roles and views of community-level participants will enable policymakers to develop more effective strategies, improving community involvement, resource distribution, and overall dengue prevention and control efforts. Thus, this study aimed to explore and provide valuable insights into the experiences and challenges faced by community stakeholders in dengue outbreak management in the urban settings of Nepal. Although multiple community stakeholders were engaged in dengue outbreak management and control, this study focused solely on FCHVs and local leaders (ward representatives), as they were the primary implementers of dengue-related activities and programs. In order to obtain an in-depth understanding of the lived experiences and perspectives of community stakeholders about dengue outbreak management, the study utilizes an interpretative phenomenological method (IPA). IPA provides a comprehensive, in-depth insight of people's subjective experiences and is ideal for understanding out how they view and interpret their roles and difficulties in dengue management.

## Method

### Study design

A qualitative study using an interpretive phenomenological approach was conducted among community stakeholders from April 2023 to June 2023. This study explored community perceptions and descriptions of their lived experiences in relation to dengue outbreak management. Our study followed the consolidated criteria for reporting qualitative studies (COREQ) guidelines to ensure standard reporting [31].

### Study site

The study was conducted in the purposively selected metropolitan city of the Lalitpur district of Nepal. During the 2022 dengue outbreak, a higher number of dengue cases were reported in Lalitpur district than in other districts, making this site more appropriate for the study [10]. The local leaders from the ward level and the FCHVs were visited at their respective workplaces and homes based on their convenience for conducting interviews.

### Sample size and sampling technique

Twenty in–depth interviews (IDI) were conducted among the community stakeholders, including the FCHVS, ward chairpersons, and ward members engaged in dengue outbreak management. The number of interviews was determined based on the data saturation level [32]. The interviews were conducted with ward representatives and FCHVs, who are the main actors responsible for carrying out dengue preventive and control initiatives at the community level. FCHVs play a critical role in health education, disease prevention, and mobilizing community action, while ward representatives are responsible for local planning, resource allocation, and coordination during outbreaks. Their direct engagement with the community and decision-making authority made them key informants to understand the experiences and challenges in dengue outbreak management. As the study concentrated on the community based experiences and issues rather than clinical perspective on dengue management, the other stakeholders in as the health care providers, public health officials were not included in the study. The participants who were not engaged in the previous dengue outbreak management were excluded during selection process.

### Data collection tools and technique

An interview guideline was designed to explore the participants'experiences and challenges in dengue prevention and control during the outbreak. The open ended questions were newly designed by the authors (SG) and (DRS) to encourage the detailed narratives, allowing the participants to share their experiences during dengue outbreak management. The guidelines were guided by a review of the relevant literature, expert consultation and study’s specific research objectives, ensuring they remained flexible to allow for probing and follow-up questions as new themes emerged during the interviews. Before conducting the interview, the enumerators identified and invited potential participants for the study. All participants who were approached in person for the interview agreed to take part in the study. The interviewers had no prior relationships with the participants, and the participants were informed about the research objectives and consent process. For data collection, a face-to-face interview approach utilising an interview guide was employed [33]. The public health professionals with a bachelor’s degree in public health and experience in qualitative data collection were recruited and provided essential training on collecting qualitative data. They were guided by one female researchers, SG (MPH, MA), and one male researcher, DRS (MSc, MA), who are experienced in conducting qualitative studies and publications.

The duration of the interviews varied, lasting between 25 and 35 min. The information was captured using an audio recording device, and field notes were taken. To improve the conformability and credibility of the data, member checking was used. This involved asking probing questions and frequently rephrasing during the interviews to confirm the accuracy of the information. After the interviews, researchers discussed the ideas shared with participants to ensure a mutual understanding of them.

### Data analysis

The recorded interviews conducted in Nepali were transcribed and translated into English by the researchers. To ensure anonymity, interviewees were assigned codes, and confidentiality was preserved by securely storing the interviews in a password-protected folder on a laptop. The cultural nuances present in the participants'expressions were carefully preserved during the translation process from Nepali to English. Instead of translating literally, a culturally conscious, meaning-based translation methodology was used. This made sure that culturally relevant references, contextual meanings, and local terminology were appropriately communicated. Because the researchers understood the local context and were competent in both languages, they were able to preserve the original intent, tone, and social realities of the participants. An independent research team member verified the English translations for accuracy and completeness. The verified English transcripts in the English language were imported in Dedoose software [34], and codes were generated. The data were analysed using thematic network analysis [35]. A thematic deductive approach [33] was applied to analyse the data. The data analysis process involved an iterative process of coding, analysing, writing, diagramming, and revising. Similar codes were grouped, and those with related meanings were merged. From the generated codes, broader themes were identified. Similar basic themes were combined to form organising and global themes, providing deeper meaning to the extracts. The thematic network analysis helped organise these themes into global themes for a thorough exploration of the specific issue (Supplementary file 1).

**Fig. 1:**
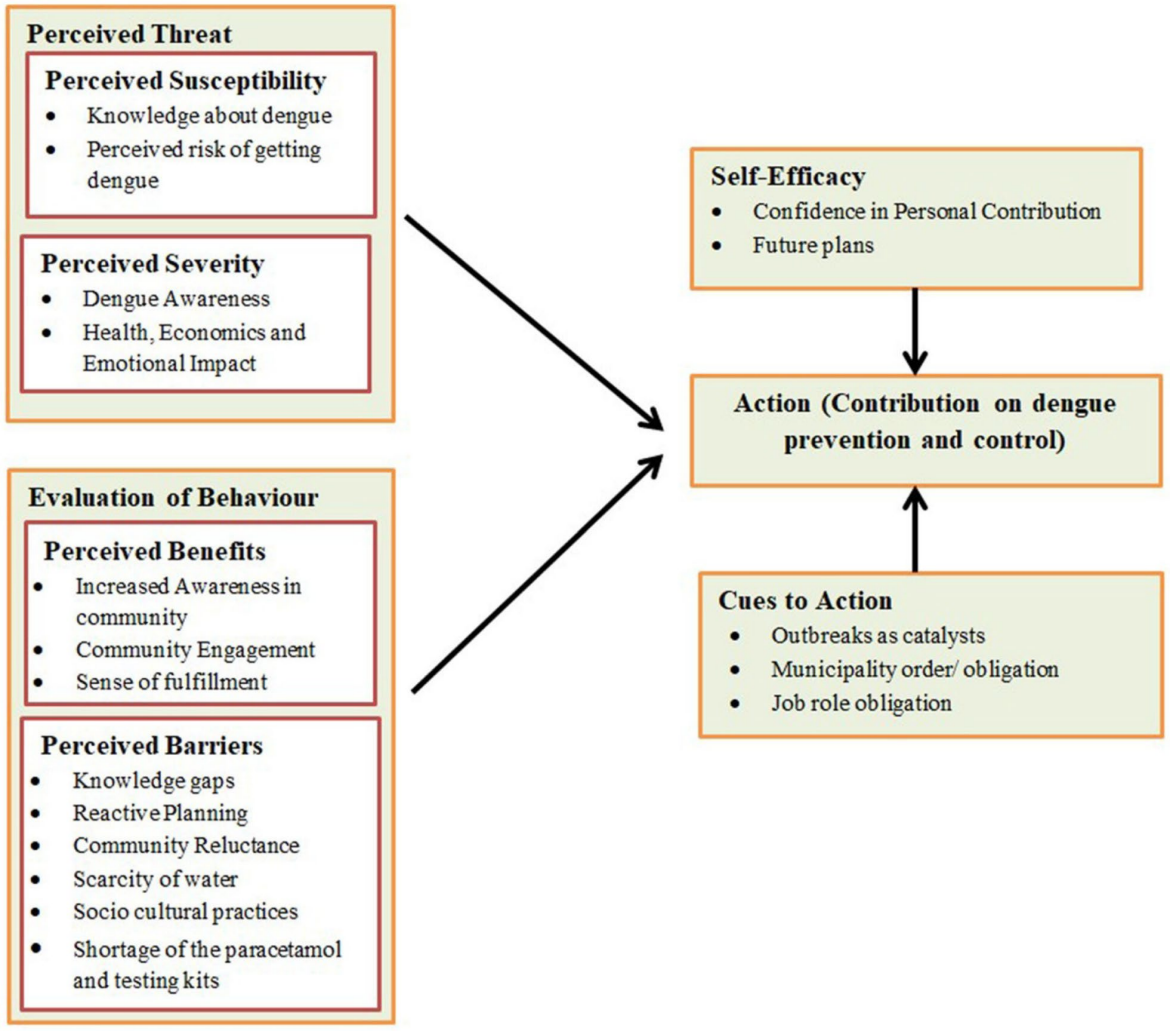
Health Belief Model presenting personal and structural factors influencing dengue prevention and control actions

The Health Belief model was used as a guiding framework for analysing the individual’s experiences with dengue prevention and management [36]. The main constructs of the model, perceived susceptibility, perceived severity, perceived benefits, perceived barriers, and self-efficacy and cues to action, were used as a lens to interpret the data. This model helped to assess how the beliefs about susceptibility to dengue and the perceived severity of the disease shape their priorities in the prevention and response to dengue outbreaks [36]. The holistic approach of the Health Belief Model is a valuable tool for analysing both personal and structural factors that impact the effectiveness of dengue control initiatives (Fig. 1). Focusing on conformability, credibility, dependability, and transferability ensured data trustworthiness [33].

### Ethical approval

The Ethical Review Board of the Nepal Health Research Council (Reference Number: 2801) granted approval to conduct this study. Written informed consent was obtained from literate participants, while thumbprints were collected from illiterate participants after the consent form was read aloud to them prior to the interview. The study adhered to the ethical review guidelines set by the Nepal Health Research Council. Participants were fully informed about the study's purpose, potential risks and benefits, and their right to withdraw at any time without needing to provide a reason. The researcher ensured the privacy and confidentiality of the data, keeping all audio recordings and translated documents secure. No personal identifiers were used in the study to protect participants'identities.

### Researcher’s reflexivity

The researchers'previous community engagement in Nepal and their public health backgrounds might have affected how they collected and interpreted the data. Peer debriefing, culturally appropriate translation, and careful interview design were used to reduce biases. Nonetheless, it is important to consider the positionality of the researchers and the intrinsic impact of their professional perspectives when understanding the results.

## Results

### Characteristics of the participants

A total of 20 key informant participants were interviewed to explore the experiences of the key stakeholders at the community level for the dengue prevention and management. Participants’ socio-demographic information is provided at Supplementary file. (Supplementary file 2). The study findings are presented under two broader themes (i) Community contributions in awareness building, and (ii) Stakeholders’ experiences on dengue management and prevention.

**(i) Community contributions in awareness building**

Community stakeholders played a crucial role in the prevention and control of dengue during the outbreak. Both the FCHVs and political leaders in the community expressed that they were engaged in the door-to-door visits to educate residents on eliminating standing water, promoting the use of protective clothing, and emphasising the importance of cleanliness to prevent mosquito breeding. One of the FCHV who has been working since the last 20 years in the community explained that;

*“As social workers, we visited homes, emptied stagnant water that had accumulated in containers and demonstrated to residents the importance of not allowing water to collect. We also informed people that water stored in drums should not be kept for extended periods.”(KII 12)*

The ward representatives further shared that along with home visits, the activity of making and distributing the posters and pamphlets was also being carried out to make the people aware of dengue. In some wards, the school awareness program and the awareness in motor workshops for the prevention and control of dengue were also found to be conducted. One of the ward chairpersons in the interview shared that;

*“Instead of fumigation, we organised a campaign where we visited households to raise awareness about potential mosquito breeding sites, such as water-filled utensils and tires found in workshops and nearby homes. We informed residents that mosquitoes breed in clean water. We have conducted many similar programs. Recently, we completed the first phase of activities, and in the second phase, we finished printing pamphlets and posters, which we plan to distribute in schools.”(KII18)*

The ward representatives further explained that the municipality had initiated the"larva search and destroy program" across the city, identifying and eliminating mosquito breeding sites like water containers during home visits. They inspected containers for larvae and discarded stagnant water. This effort extended beyond homes to include garages, halls, and neighbourhoods, where they targeted and destroyed mosquito pupae and larvae. A ward member in the community in an interview explained that;

*“Last year, we launched a program called "Search and Destroy" in Lalitpur Metropolitan City. As part of this initiative, we inspected Ward 4, checking flower pot plates, discarded utensils, pooled water from washing dishes, and other mosquito breeding sites. The "Search and Destroy" program was implemented across all wards of Lalitpur Metropolitan City. FCHVs, the health workers and health facilities, ward members, and other stakeholders were involved in carrying out these activities.” (KII20)*

The study participants recognised significant benefits from collaborative efforts in dengue prevention and control at the community level. The coordination between various stakeholders, including FCHVs, Tole Health Promoter (THP), the Tole Promotion Committee, the Ward Health Committee, municipality officers and political leaders from the ward, was seen as crucial in organising effective awareness programs and mobilising the community. One of the FCHV who served to the community since last 16 years has stated that;

*“We along with ward members, THP (Tole Health Promoter), members from WHC (Ward Health Committee), went to provide awareness in the community with the coordination of ward.”(KII13)*

These collaborations with Urban Health Clinics and the ward facilitated proactive measures such as home visits and awareness campaigns before outbreaks. The health office’s involvement further strengthened these initiatives. Stakeholders perceived these joint efforts as instrumental in enhancing community engagement and maintaining a dengue-free environment, with a strong belief that continuous improvement by the metropolitan authority would yield even better results. A ward chairperson in the community stressed that

*“… We organised the meeting at the ward with the health professionals at UHC to discuss how we can prevent and control dengue. Then, we develop plans and execute them through various groups, such as the Tole Promotion Committee within the community. We liaise with the president of the Tole Promotion Committee to coordinate their role in facilitating household visits aimed at raising public awareness about dengue prevention and control measures in that specific area.”* *(KII 16)*

The community’s collective efforts greatly contributed to reducing dengue outbreaks and raised awareness, showcasing the effectiveness of grassroots public health initiatives.

**(ii)** Stakeholders’ experiences on dengue management and prevention

The experiences and challenges faced by the community stakeholders during the dengue outbreak are presented under these specific themes guided by the key components of the health belief model.

- **Perceived susceptibility**
- Knowledge about dengue

The participants have similar perceptions and in-depth knowledge regarding the causes, mode of transmission, common symptoms, and preventive measures of dengue fever. The FCHVs who work under the health system and have relationships with the households and the individuals in the community have provided correct information regarding dengue. One of the FCHVs, who has been working in the community for 16 years described the dengue as;

*“Dengue is seasonal and is spread by the Aedes mosquito. To prevent this disease, we have to maintain cleanliness around our houses; we shouldn’t let water be collected in containers, unwanted shrubs should be trimmed, and disposal of collected or standing water should be done; this mosquito is active after sunrise and before sunset. This is spread by the bite of female mosquitoes.” (KII13)*

Similarly, the political leaders in the community, who were engaged in the dengue management and control program, were also well-informed about dengue fever. A ward member in the community explained the dengue as.

*“This disease usually spreads during summer and rainy seasons, and it can lead to death as well…… This is caused by mosquitoes. Mosquitoes breed in clean standing water, and they lay eggs over there. Compared to last year, cases are yet to rise this year. For this, we have to prepare in advance.” (KII 20)*

The stakeholders in the community who are responsible for implementing the dengue prevention and control initiatives have an adequate and strong understanding of dengue. They have received information from a variety of sources, including the news media, awareness and training, and personal experiences in managing the dengue outbreak.

(b) Perceived risk of getting dengue

The participants in the study were well aware about the risk of getting dengue. They recognized that the widespread infection in their area posed a risk both to individuals and the larger community. Training and orientation programs obtained from the Municipality and District health offices heightened their understanding of this risk, reinforcing their perception of susceptibility on both personal and community levels.

The Female Community Health Volunteers (FCHVs), who engage in house-to-house visits and participate in dengue control activities, believe that they are personally at risk of contracting dengue due to the nature of their work, which increases their exposure to the virus. One of the FCHVs with 26 years of experience in the community shared her perception and experiences as;

*“As everyone is at risk, everybody in the society should stay protected, they should wear mosquito repellent creams, full sleeved dresses, mosquito repellent coils should be burnt……….Yes, I suffered from dengue during house-to-house visits. I was walking in the rally and in the search and destroy program. In walking in the rally of 4 days, I might get bitten by a mosquito on the last day, due to which I get infected. It was so measurable; I felt like I was unconsciousness. My bones pained like it was broken.” (KII 15)*

Similarly, the political leaders in the community have similar perceptions regarding the risk of contracting dengue. They are well aware that the whole community is susceptible to infection. One of the leaders as ward chairperson in his interview described that.

*“This disease (dengue) is not new in our area, especially during the rainy seasons………Last year in our ward only, we had 551 cases of dengue.” (KII 18)*

Thus, a strong perception of susceptibility was observed among the responsible community stakeholders in the community. Their sense of vulnerability is evident as they acknowledge that everyone in the community is at risk, especially when directly engaging in activities that increase their exposure to mosquito bites. Despite acknowledging the risks, all stakeholders maintained a belief that dengue is preventable with proper measures.

- **Perceived severity**
- Dengue experiences

Participants vividly described the severity of dengue fever through their experiences with a spectrum of symptoms such as severe headaches, vomiting, diarrhoea, high fever, body pain, and loss of appetite. These symptoms frequently led to hospitalisation, where treatment included saline and medications like paracetamol and Oral Rehydration Solution (ORS). One FCHV with 18 to 20 years of experience in the community, shared:

*"This is a dangerous disease which affects more people… I myself had dengue. I was about to die. I went to the hospital, and they gave me saline water."* (KII12)

The perception of dengue's severity extended beyond physical symptoms, with participants also describing psychological distress. Some mentioned fears of dying, while others were debilitated to the point of being unable to perform basic tasks, such as walking. The ward member in the community also said that;

*"I have been bedridden for 27 days… It was so miserable."* (KII17)

The study participants had a heightened perception of dengue's severity due to their varied experiences, ranging from mild to severe symptoms. These experiences emphasised the serious and unpredictable nature of the disease, with some viewing it as life-threatening and others finding it debilitating enough to hinder basic activities.

(b) Health, economic, and emotional impact of dengue fever

Dengue was perceived as a serious issue with significant emotional and financial consequences, indicating a high level of perceived severity among the community. The emotional distress was evident, with some participants feeling near death, while others experienced severe physical debilitation that affected their ability to perform daily tasks. One FCHV serving the community for 18–20 years shared that;

*"At first, I had a severe headache, vomiting, diarrhoea, and then I got a fever and severe body pain. I couldn't even walk; my sisters had to take me to the washroom."* (KII 12)

In addition to the physical challenges, the financial burden was substantial due to hospitalisations and extended recovery times. Participants discussed the strain of prolonged illnesses, which often required costly medical treatment and left them unable to work or engage in normal activities for an extended period. A FCHV having 18–20 years of experience in her interview explained that;

*"There are two people in our family, and both of us caught dengue. I stayed in the hospital for five days. I also had a urine infection, so I was even weaker."* (KII 14)

Some participants described complications such as liver and urinary infections, exacerbating their health struggles. They perceived that the infection not only worsened their physical condition but also contributed to their belief that dengue was more dangerous than other diseases, including the COVID-19 pandemic. Others shared the impact of being bedridden for extended periods due to dengue, underscoring the debilitating effects of the disease on their everyday lives.

A ward representative who serves as a ward member in the community stated:

*"This disease spreads during summer and rainy seasons and can lead to death. Last year’s epidemic showed that this disease is more dangerous than COVID-19."* (KII 20)

Further emphasising the high perceived severity, many FCHVs expressed concerns over the risks they faced while conducting programs. They noted that despite the dangers, they lacked health insurance, which heightened their vulnerability during outbreaks. An FCHV serving the community since last15 years said:

*"Last year when many of the FCHVs contracted dengue while conducting programs, they couldn’t do anything. We don’t even have health insurance. We have to take risks ourselves and work to provide social service."* (KII 2)

The community stakeholders’ perception of the severity of dengue fever is deeply rooted in their personal and shared experiences of physical suffering, emotional distress, and financial burden.

- **Perceived benefits**
- Increased awareness

Community stakeholders perceived that increased awareness levels in the community have been one of the most significant benefits of their efforts in dengue prevention and control. They observed that people had started adopting preventive measures, such as disposing of stagnant water and covering water containers with lids. One of the FCHVs working in the community for the last 20 years noted that;

*"People have started using prevention techniques now a days. People have disposed of collected water in plates of pots, and water is being covered with lids. People have been more aware now."* (KII 14)

The shift in behaviour reflects a substantial change in community attitudes. Individuals who were once indifferent have now become more vigilant, especially after witnessing the impact of a dengue epidemic. Initially, there was fear surrounding the disease, as one ward chairperson explained,

*"Initially, people were scared by the name of dengue. But now they are being aware and understand what it is…"* (KII 16)

These government-led and FCHV-driven awareness programs have significantly enhanced awareness, fostering a proactive approach to dengue prevention and control. This collective effort has been instrumental in shifting perceptions and behaviours to promote greater community resilience.

(b) Community engagement

Community stakeholders perceive increased community engagement as a significant benefit of their collaborative efforts in dengue prevention and control. This is evidenced by the proactive behaviours observed during follow-up visits, where people began disposing Aedes mosquito breeding sites on their own after initial guidance.

One of the political leaders as a ward chairperson with higher education explained that;

*“We have 16/17 FCHVs, Health Facility and Management committee, Women’s group in the community, clubs and Tole Promotion Committee, we all work collectively and all search, verify and destroy the larva. We have at least one member from the ward representative actively getting involved in the awareness and search and destroy program… Eventually, we formed a team consisting of women's groups, FCHVs, health workers from Ward ‘X’, and ward volunteers. Together, we carried out community miking and conducted house-to-house visits to search for and destroy larvae in water tanks, plastic bags, and other household items. After implementing this program, we saw no cases this year.” (KII 18)*

The continuous implementation of various prevention and control programs has further solidified community involvement, demonstrating a sustained commitment to combat dengue and highlighting the success of these collaborative efforts in fostering active participation from community members. One of the FCHVs, with seven years of experience in interviews, observed that;

*“Following our initial home visit, by the second visit the following year, people had begun independently eliminating Aedes mosquito breeding sites.” (KII1)*

The community’s active participation, facilitated by collaborative efforts, has been instrumental in reducing dengue cases and demonstrates the effectiveness of engaging local stakeholders in long-term prevention and control initiatives.

(c) Sense of fulfilment

The FCHVs in the community perceive a strong sense of self-satisfaction and fulfilment as one of the key benefits of their involvement in dengue prevention and control efforts. This feeling is rooted in the recognition that providing awareness and contributing to public health is both as a duty and a form of social service, which gives them a deep sense of pride and meaning in their work. For many long-serving FCHVs, the experience of fulfilling this role over the years has only deepened their sense of purpose. One who worked as a Tole health promoter at the beginning and has been working as a FCHV since three years explained that,

*“I have been FCHV for a very long time. I feel so good while doing this job because to do social service without earning much is a good deed.”* *(KII 1*)

However, although the local political leaders in the community were regularly engaged in dengue prevention and control efforts, none of them expressed a sense of fulfilment as a perceived benefit of involvement in dengue prevention and control.

The sense of personal fulfilment and intrinsic satisfaction derived from these collaborative efforts in dengue prevention and control not only motivates community volunteers but also sustains their long-term commitment to public health service.

- **Perceived barriers**
- Knowledge gaps in the community

Local leaders and health workers recognise a significant knowledge gap in the community as a critical barrier to effective dengue prevention. Although some awareness has improved, particularly after dengue outbreaks, the government’s reactive approach, focusing on awareness only after an epidemic has started, hinders long-term prevention efforts.

One local leader emphasised the community's lack of knowledge, explaining that many people continue to engage in risky behaviours, such as collecting water in open containers. This creates ideal breeding sites for mosquitoes. The local leader, as a ward member, pointed out,

*“People in the community collect water in containers due to lack of knowledge. In most cases, people who reside in rented housing have a tendency to store water in various types of open containers where mosquito eggs and larvae are found, spreading in the community.” (KII17)*

The issue of delayed awareness campaigns was further highlighted by an FCHV, who reflected on how, in the past people were dismissive of dengue-related warnings. However, the situation changed only after experiencing a dengue epidemic. The FCHV explained,

*“A few years ago, when we told people in the community about dengue, they ignored it; they weren’t aware. But after the epidemic, people became more aware. The Government also conducts awareness programs by using FCHVs after the epidemic but doesn’t do anything beforehand.” (KII 4)*

This reactive approach undermines the potential for pre-emptive action and leaves communities vulnerable to outbreaks. Although awareness about dengue has improved, particularly post-epidemic, the gap in proactive education and the reactive stance of the government contribute to ongoing challenges in effectively managing and preventing the disease in communities.

(b) Reactive planning mechanism

Local leaders and health workers perceive the reactive planning approach of the Government and local authorities as a major barrier to effective dengue prevention. Awareness programs, often conducted by Female Community Health Volunteers (FCHVs), are typically initiated only after outbreaks have begun, which undermines efforts to prevent the disease from spreading. Leaders stressed the importance of launching awareness campaigns before any cases appear to halt the spread of dengue from the outset.

One FCHV, with 13 years of experience working in the community, shared their frustration, explaining that while they conduct health camps and awareness as trained, there are no proactive programs in place.

*"Recently, there haven’t been any programs for this year; programs happen only after the outbreaks. The Metropolitan authority conducts these programs only after outbreaks have started, but they should be conducted beforehand (before outbreaks)."(KII 4)*

A ward chairperson also described how reactive the current system is, explaining that dengue prevention and control measures only begin once a few cases have been reported.

*"Once we learn about 1 or 2 cases of dengue, we move towards implementing prevention and control measures, such as cleanliness drives, search and destroy programs, and public awareness through household visits."(KII 16)*

The reactive nature of government and local authority planning, coupled with insufficient support for health workers, hampers effective dengue prevention. Timely proactive measures, such as early awareness campaigns and better support for frontline workers, were believed to be necessary to mitigate the spread of dengue and protect both the community and those working to prevent the disease.

(c) Community reluctance

Community reluctance has emerged as a significant barrier to dengue prevention and control efforts, as local leaders and health volunteers encountered resistance during their campaigns. Despite efforts to educate residents, many dismissed the seriousness of dengue or mistrusted the government's intentions, particularly in wealthier households. This resistance made it difficult for health volunteers to implement preventive measures like disposing of mosquito breeding sites.

One of the FCHVs, having 15 years of experience, explained the challenges faced during home visits, sharing that residents often refused to engage.

*"Though we had ID cards, people thought we were there to collect donations and refused to open their doors. Domestic dogs even chased us. These were the challenges we faced*.*"(KII2)*

This resistance was particularly evident in affluent areas. Another FCHV who has worked in the community for 26 years, shared that,

*"In VIP (affluent) areas, well-educated and wealthy families, including doctors, would tell us,'We are capable ourselves, and so you don’t need to say or do anything in our home.'They didn’t let us enter, but later, we found those same households getting infected."(KII15)*

Ward representatives as a ward member in the community also feel the same experience as;

*“Yes, it is very difficult to convince the people in the community. Despite raising awareness about the risks of dengue, they won’t remove the water themselves, we have to forcibly destroy it.” (KII 19)*

The reluctance of the community to engage, particularly among affluent residents, along with mistrust in government initiatives, presents a major obstacle to dengue prevention efforts. This resistance not only frustrates health workers but also undermines the effectiveness of public health campaigns, leading to continued vulnerability within the community.

(d) Scarcity of water in the community as a barrier for dengue prevention

Water scarcity in the community emerged as a significant barrier to dengue prevention and control efforts, as local stakeholders struggled to balance water conservation and storage with mosquito control. In rented households, the limited water supply especially forced residents to store water in open containers, creating ideal breeding grounds for mosquitoes. Health workers encountered resistance when trying to eliminate larvae from these containers, as residents prioritised the preservation of water over dengue prevention.

One FCHV working for the last 18 to 20 years in the community has described the challenges they faced, saying,

*"More than opportunities, we faced many challenges. In Kathmandu, where there is a scarcity of water, when we tried to dispose of water, we were scolded with remarks like,'Will you come and fill these drums?'Some even acted as if they were going to beat us. Even though government officials were with us, people didn’t listen, and we had to revisit those houses with the police."(KII12)*

Another stakeholder working as a ward member emphasised the complexity of convincing residents, sharing,

*"When we go to destroy the larvae from the container, they won’t let us because of the water shortage. They use excuses, saying they need that water for household purposes, but we convince them and destroy it. This sometimes leads to conflicts and disputes."(KII16)*

The severity of the water scarcity problem was further explained by another local leader who served as a ward member in the community,

*"The first challenge is the shortage of water in the community, especially for the rented population. Water is supplied only once a week in very limited amounts, around 1000 litres, which is insufficient. So, they accumulate and store water in whatever containers they have, open or closed, and collect rainwater in open drums, which provides favourable conditions for mosquitoes to breed."(KII17)*

The tension between the community’s need for water storage and the efforts to control mosquito breeding presented a significant challenge. Despite health workers'efforts to educate the community about the risks, water scarcity remained a persistent issue that complicated dengue prevention measures, at times necessitating the involvement of authorities to enforce compliance.

(e) Socio-cultural practices as a barrier

Local leaders and health workers identify certain cultural practices as barriers to effective dengue prevention. Traditional customs, such as storing old utensils and keeping water in religious vessels like the"*Kalash*,"inadvertently create environments conducive to mosquito breeding. Despite awareness efforts, these practices persist, undermining the efforts to control the spread of dengue. One ward member in the community pointed out how cultural habits contribute to the problem, stating,

*"This disease is also caused by our cultures and traditions. People have the practice of storing water in utensils, which allows mosquitoes to breed in those utensils."(KII15)*

Another stakeholder, a ward chairperson of the community, highlighted specific community practices, sharing,

*"In our community, there's a practice of keeping water on rooftops for birds and in utensils for dogs, where we found larvae."* *(KII18)*

FCHV worked as a Tole health promoter for four years, and FCHV, since the last three years, had also noted similar challenges and described as,

*"We go from one house to another and tell them to remove the water from plates of flower pots. They remove the water, but the next day, when we visit their house again, there will be water in the'Kalash'(a religious vessel where water is kept). In my house, there is no water in the Kalash."* *(KII1)*

However, not all cultural or religious beliefs seem to be direct barriers to dengue prevention. One FCHV having more than 18 years of experience in the community have shared that,

*"People in the society think that dengue is caused by mosquitoes. There aren’t any religious or superstitious ideologies related to dengue."* *(KII14)*

Although no direct religious or superstitious beliefs impede dengue prevention efforts, certain cultural habits, such as storing water in vessels for religious or practical purposes, remain significant barriers. These observations highlight the need for culturally sensitive approaches in designing public health interventions to address such practices effectively.

(f) Shortage of paracetamol and testing kits

The lack of essential resources like paracetamol and dengue testing kits was a major obstacle in managing the dengue outbreak, as described by community stakeholders. As the number of cases surged, the demand for these critical supplies quickly exceeded availability, largely due to inefficiencies and corruption within the healthcare system.

One ward chairperson highlighted the issue, explaining,

*"I've also discussed the availability of paracetamol with the health team at the Urban Health Clinics. In our country, corruption is so rampant that as soon as news about dengue spreads, there's often a shortage of paracetamol and dengue kits. To prevent this, I've requested that our ward stock up on paracetamol to distribute when needed."(KII18)*

The shortage of paracetamol, a basic but essential medication for managing fever and pain, caused widespread anxiety among community members and delayed treatment. Similarly, the lack of dengue testing kits hindered accurate diagnosis, making it difficult to monitor and manage the outbreak effectively.

Despite local health teams’ efforts to secure additional supplies, the persistent shortages of paracetamol and dengue testing kits exposed systemic flaws in the healthcare infrastructure during outbreak. These challenges significantly weakened the community's response to the dengue crisis, illustrating the need for better resource management and transparency in public health efforts.

- **Cues to action**
- Outbreaks as catalysts

The surge in dengue cases acted as a powerful motivator for community stakeholders to quickly implement preventive and responsive measures. Initially, with no cases reported in the previous year, there was consideration of reallocating the budget away from dengue prevention. However, once new cases were identified in nearby wards, stakeholders promptly resumed their plans to address the outbreak. Resources from the disaster management budget were reallocated to support dengue prevention activities, emphasizing the seriousness with which these stakeholders responded to the looming threat.

As one ward chairperson explained, *"We used to allocate the dengue under the health. And in this fiscal year as we spent the entire budget, we were confused about whether to allocate a budget as no dengue case was reported the previous year. But once we heard of dengue cases in the nearby ward, we planned to conduct and continue the previous activities in dengue prevention and control. The budget under disaster management was allocated for this activity."(KII18)*

The anticipation of increasing dengue severity, particularly the risks associated with reinfection, increased stakeholders’ vigilance. They proactively discussed these concerns in ward meetings, ensuring that home visits and other prevention measures would be conducted if the disease spread within the community. This proactive approach underscored the importance of staying prepared even during periods of low infection.

An FCHV who worked as a Tole health promoter for four years and has been working as FCHV for the last three years noted that,

*"We talked in the ward about this disease in the meeting. We have heard that this year the severity and epidemic of the disease will rise as compared to last year, and also we have heard that once an individual is infected and gets re-infected again, then the complications of dengue rise more in that individual."(KII1)*

Another FCHV having 16 years of experience reinforced the readiness of community health workers, sharing that,

*"This year also, if dengue spreads in our community, we’ll go for home visits."(KII13)*

The spike in dengue cases acted as a cue to action for stakeholders, triggering rapid resource mobilisation and preventive strategies. This prompt reaction demonstrated the community's commitment to safeguarding public health in the face of the ongoing threat of dengue.

(b) Municipality order/obligation

Municipality and ward directives acted as essential cues to action for local stakeholders, prompting their active engagement in dengue prevention and control. The involvement of higher authorities played a crucial role in mobilising community members and health workers to implement preventive measures, such as home visits, awareness programs, and counselling sessions. These orders ensured that the community remained proactive in combating dengue outbreaks by creating a structured framework for action.

For instance, one Tole health promoter working in the community since last 10 years explained,

*"We provide awareness about dengue to community people as directed to us by the ward."(KII 8)*

This directive-based approach led to the implementation of awareness campaigns, with volunteers visiting homes, distributing pamphlets, and providing guidance on dengue prevention and treatment.

Another health volunteer, described the comprehensive nature of these efforts, saying,

*"From the public health section of the metropolitan city, we were utilized to provide awareness to people by visiting their homes and distributing pamphlets and also providing counselling to prevent the disease. We used to go 2 h in the morning and 2 h in the day for this program. During the day we also used to visit 1 h per day and counsel people in their respective homes about dengue, its prevention, and treatment. From the ward level, we did miking about dengue in many junctions, gathered people, did miking for some time, and moved to another junction."(KII2).*

In addition, the consistent support and involvement from the municipality's public health section encouraged local stakeholders to sustain their dengue prevention efforts. As one ward chairperson noted that;

*"At the municipality level, there is a health committee and also a public health section at the municipality. They regularly visit here recently they also came here to conduct an awareness program and regularly encourage us to conduct the dengue management and control measures."(KII18)*

These orders and obligations from higher authorities acted as significant triggers, ensuring that the community maintained vigilance and took the necessary actions to manage and control dengue outbreaks.

(c) Job role obligations

Job role obligations served as key cues to action, motivating community stakeholders to actively engage in dengue prevention and control. These obligations, reinforced by regular orientations and expectations to conduct home visits, ingrained a sense of responsibility among health workers. As a result, they often found themselves addressing dengue-related issues even outside formal duties, demonstrating their deep commitment to public health.

A participant, who was a ward representative, explained,

*"We will receive orientation again soon about home visits and we’ll perform home visits this year as well. Now, it has been a habit for us to be aware of these things even though we haven’t started home visits. If we see people collecting water with larvae, we suggest they dispose of it."* *(KII9)*

This proactive behaviour illustrates how their roles have become second nature, ensuring they remain vigilant in promoting dengue prevention even beyond scheduled activities.

Involvement in official programs further emphasised their commitment. Another participant noted,

*"The health office has some programs, so we’ll be involved in that program as well."* *(KII6)* Despite facing personal hardships, including contracting dengue themselves, the obligation to fulfil their job roles persisted as a powerful motivator. One of the Community health volunteers shared the challenges faced by saying,

*"People in the community say that we get paid for doing these works, but being a social worker we only do social service. There should be some facility for us. During the conduction of these programs, we ourselves got dengue. Our incentives should be raised because we do work for the sake of the community. We must be available at all times—there is no fixed schedule. Whenever we get a call, we have to leave whatever we’re doing and be present in those areas, which sometimes make it’s difficult to reach the location."* *(KII14)*

Despite the lack of incentives and support, the stakeholders remained dedicated to their roles, driven by the sense of duty to safeguard the health of their community. This sense of obligation ensured their consistent involvement in dengue management, highlighting the importance of their contributions.

- **Self-efficacy**
- Confidence in personal contribution

Confidence in their personal contributions was a key aspect of self-efficacy for community stakeholders, driving their continued engagement in dengue prevention and control. Their belief in the effectiveness of their actions was evident through their proactive initiatives, such as conducting awareness programs and home visits, reflecting their confidence that these efforts could reduce dengue cases. Despite the challenges they faced, stakeholders remained steadfast in their resolve, continuing their work even without significant support from higher levels of government. This sense of self-efficacy reinforced their commitment to combating dengue and addressing public health issues within their community.

One ward representative as a ward chairperson expressed this confidence, stating, *"Rather than giving them some suggestions, we act on our own efforts from the ward and municipality. If there was no local government, how would they face the public? There is no presence of the central government either in developmental works or during outbreaks like dengue."(KII18)*

The Female Community Health Volunteers (FCHVs) also expressed a similar sense of determination and confidence. One volunteer who had been working as an FCHV for the last 18–20 years expressed her confidence as,

*"Challenges come and go. We tackle those challenges and move forward."(KII11)*

This collective confidence in their contributions enabled stakeholders to persist in their roles, maintaining their commitment to public health despite systemic challenges.

(b) Future plans

Their future plans strongly reflected their self-efficacy, showcasing their confidence in their ability to make a positive impact on dengue prevention and control. Their proactive approach included conducting awareness programs ahead of potential outbreaks to ensure timely education and community preparedness. As discussed during their meetings with the ward, they demonstrated a commitment to pre-emptive action. For instance, one Female Community Health Volunteer (FCHV) with 13 years of experience expressed that,

*"We’ll raise awareness as per our training, conduct free health camps for dengue check-ups….Our team has been discussing conducting an awareness program before the outbreak starts so that timely awareness will be provided. We have been talking with the ward for timely conduction of the program"(KII4)*

A ward representative who was the ward chairperson also outlined specific, actionable steps, saying,

*"Yes, cases have been reported in Wards 5, 2, 4, and in either Ward 11 or 12, so I’ve instructed the printing of banners, which should be completed by now. Our next step is to move into the Toles in this ward, display dengue-related banners, distribute posters and pamphlets, and carry out house-to-house visits to implement the public awareness program and search and destroy program… We're focusing on public awareness, and I've allocated a budget for it. I’ve also discussed the availability of paracetamol with the health team at Urban Health Clinics."(KII18)*

Their strategies highlighted a collective belief in their ability to make a meaningful difference in controlling dengue, demonstrating a commitment to proactive measures that could enhance community health and safety.

## Discussion

The study aimed to assess the experience of community stakeholders involved in the prevention and control of dengue outbreaks using the Health Belief Model as a guiding framework in the urban area of Kathmandu Valley. The study revealed a crucial comprehension of community stakeholders’ perspectives, difficulties, and aspirations navigating the complexities of dengue prevention and control.

Perceived severity and increased susceptibility are important motivators for taking health related action. The stakeholders conveyed that the community's recurrent outbreaks contribute to a high perceived susceptibility and severity. The perception of dengue's susceptibility and severity emerges from awareness of the disease's threat, personal experience with it, and careful observation of its consequences and complications in the community among community stakeholders. Unlike the study where the perceived susceptibility was linked to the mosquito density [37]. The current study results were in line with a previous study that showed populations exposed to dengue exhibit elevated levels of perceived susceptibility and a greater awareness of the severity of the disease [38]. Another study in Malaysia and Pakistan also demonstrated that having adequate knowledge, perception of the dengue’s severity and susceptibility differed greatly depending on their personal experiences, with those who had close observation of fatal dengue cases perceived the disease as very serious and felt extremely vulnerable [39–41]. The good knowledge among the community stakeholders might be due to training and the orientation programs they received in order to conduct the awareness campaign in the community and regular engagement in the dengue prevention, control and management initiatives. However, findings from a qualitative study conducted in Australia revealed that perceived community indifference contributed to stakeholders'frustration in mobilizing community participation in vector control and preventive efforts [42].The study found that during the community's dengue outbreak, the involvement of community stakeholders was vital for the prevention and management of the disease. As a result, the community leaders viewed their efforts as beneficial, including their home-to-home visits, public awareness programs, search and destroy campaigns, and collaborative initiatives between the municipality and the locals. Their efforts ultimately resulted in a rise in community awareness, community involvement in dengue prevention and control, and self-satisfaction. Therefore, the community stakeholders perceive this approach as having fostered a sense of responsibility in the community, which has been beneficial. Similarly, studies in different countries also revealed that community-based initiatives have been applied and are expected to be effective for dengue prevention, control and eradication [43, 44]. Likewise, another studies also highlighted the importance of the partnership between the community and authorities for better management and control of the dengue outbreak [45, 46]. However, the population in Malaysia believed that preventive measures might not reduce the risk of getting dengue, which resulted in a lack of perceived benefit for efforts aimed at prevention and control [40]. Also in some communities, the community perceived that the dengue prevention and control efforts are the sole responsibility of the Government [37].

As a paper critically analysing dengue prevention and control through the historical experiences of six countries, it emphasizes that although community-based efforts are crucial and beneficial, they remain insufficiently effective due to the complexities of the socio-ecological system [44]. Similarly, in the current study, community stakeholders also face several challenges during implementation. The low level of knowledge in the community is one of the identified barriers by the community stakeholders in this study which is common and reported in the various studies [41, 47, 48]. The reactive planning (delay) approach from the higher authority is also a barrier for the community stakeholders in which the community already gets infected and is at risk. A previous study conducted in Nepal also reported delays in awareness campaigns as a limitation of the dengue outbreak response [49]. Similarly, a study also explained reactive planning as a challenge, suggesting the shift towards proactive approaches for better dengue management and control [50]. Another study also revealed that the initiatives and the control efforts were only conducted after the outbreak, leading to a decrease in the effectiveness of the program [37]. Similarly, a qualitative study from Brazil indicated that governmental delayed and inadequate responses to dengue outbreak management contributed to a decline in community confidence [51].

Community reluctance is also a barrier to source reduction, in line with other studies which also admitted the challenge of constantly keeping the environment clean and destroying breeding sites [40, 44]. Also a qualitative study from Vietnam identified the low readiness from the community as the barriers to implement effective community engagement [52]. Similarly, another study conducted in Sudan highlighted that, alongside delayed responses, weak community engagement and community behaviours posed significant barriers for the health promotion department [53]. The lack of regular water supply has been identified as a key challenge by the community stakeholders, which is why the community people have a tendency to store water, making breeding sites favourable for the mosquitoes. This barrier was also observed in a study conducted in Nepal [49]. Similarly, a study in Vietnam also identified that improved water supply is critical for effective dengue prevention and control [54]. Also a study conducted in India identified the poor infrastructure as intermittent water supply as a barrier perceived by key stakeholders involved in the dengue prevention and control [55]. Comparably, the community's susceptibility was increased by the scarcity of testing kits and paracetamol, which made it more challenging to appropriately detect cases and manage symptoms. Another study also reported that due to the unavailability of treatment for dengue, the community relies on traditional medicine [40].

Various studies reported the traditional and superstitious beliefs that dengue occurs due to bad luck, chance, fate, or uncontrollable factors and involves scratching the body with a coin to cure illness; regarding dengue, which could act as a barrier for dengue prevention and control activities [40, 56]. However, this study doesn’t show such beliefs; rather, socio-cultural practices like placing water for the dogs and birds in the roof acted as a barrier as this water also serves as a potential breeding site for the dengue mosquito.

The current study found that the spike in dengue cases in the community or in the nearby community is a cue to action, which is in line with the study conducted in Malaysia [57]. Furthermore, orders from higher authority and their job role obligations are external cues to action. Another study in Malaysia also showed high cues to take action among the participants [58]. Similarly, an exploratory study conducted in Australia also showed the normative factors as organizational norms and ethical obligations driving the community engagement for dengue prevention and control [42]. However, a qualitative study carried out in Vietnam showed that the community stakeholders lacked dedication, which led to them not promoting their responsibility in the dengue vector control program [52].

The community leaders'self-efficacy for dengue preventative and control measures was demonstrated by their intentions for the future and their confidence in contributing to the community. This is a very important Health Belief Model construct. This result is in line with a study that stressed the value of awareness campaigns for long-term behaviour and the development of self-efficacy [58].

## Limitations and strengths of the study

Although the study provides valuable insights into the experiences of the community stakeholders in implementing dengue prevention and control efforts and strategies, several limitations should be acknowledged. Firstly, as an exploratory qualitative study with purposive sampling in the urban context, the findings could not be generalised to other rural settings. Secondly, the limitation in participant selection was the exclusion of high-level stakeholders and policymakers in the government. The study sample included only the key implementers from the ward level. It is important to recognize that the inclusion of ward leadership as participants may have introduced a social desirability bias, wherein individuals may have been less inclined to disclose shortcomings or criticisms related to their own ward’s dengue control efforts. The absence of community residents as participants may have limited the diversity of perspectives captured. Therefore, the study findings should be interpreted with consideration of this potential bias. In addition, the dominance of FCHVs in the study, while potentially introducing bias, is justified due to their crucial role in dengue prevention and control efforts, making their insights essential in shaping effective public health strategies.

Despite these mentioned limitation, the study presents an in-depth understanding of the experiences and perceptions of the implementing actors of the dengue prevention and control strategy, which fills a gap in the relatively scarce literature on the influence of the Health Belief Model on dengue in the dengue in endemic regions.

## Conclusion

This study, using the Health Belief Model, explored the experiences of some community stakeholders involved in dengue prevention and control in an urban area of the Kathmandu Valley. The study found that perceived severity and susceptibility, heightened by recurrent outbreaks, were key motivators for action. Community-based initiatives, such as awareness programs and collaboration with authorities, were seen as effective but were hindered by challenges like reactive planning, low community knowledge, reluctance for sustained efforts, and inadequate water supply. Despite these barriers, stakeholders demonstrated strong self-efficacy and commitment to future dengue prevention efforts, underscoring the need for continued education, proactive planning, and resource support to enhance community-led prevention efforts. The study emphasizes that in order to enhance dengue prevention efforts, community-based engagement, capacity building, and resource allocation for frontline community stakeholders must be strengthened. It recommends that in order to guarantee prompt and efficient outbreak responses, public health policies should encourage community involvement, offer sustainable funding, and more effectively integrate ward-level leadership. Additionally, removing operational challenges that stakeholders have pointed out can improve the efficacy of subsequent interventions and ensure a more prompt, well-coordinated, and context-specific public health response during dengue outbreaks.

## Supplementary Information

Additional file 1. Table S1. Thematic network analysis framework

Additional file 2. Table S2: Socio-demographic information of the research participants

## Data Availability

The data used and/or analyzed during the current study are available from the corresponding author upon reasonable request.
