# Supplementary material for: A qualitative exploration of community stakeholders perspectives on dengue outbreak management in urban Nepal: navigational insights and challenges
Source: Trop Med Health. 2025 May 30;53:77. doi: 10.1186/s41182-025-00758-w (PMC12123987; doi:10.1186/s41182-025-00758-w)
Supplement: Supplementary file 1 — Additional file 1. Table S1. Thematic network analysis framework [file 41182_2025_758_MOESM1_ESM.docx]

**S1 Table: Thematic Network Analysis Framework (from codes to global themes)**

| **Codes** | **Basic themes** | **Organizing themes** | **Global themes** |
| --- | --- | --- | --- |
| - Knowledge on dengue - Knowledge on sign and symptoms - Knowledge on treatment - Knowledge on vector and transmission - Knowledge in Preventive activities - Attitude towards dengue - Dengue outbreak in community - Dengue in family - Infected with dengue - Experience of being ill - Impact of dengue in daily life - Emotional impact of dengue - Home to home visit - Awareness campaign - Poster and Pamphlets distribution - School awareness program - Fumigation - Search and Destroy - Collaboration from ward and municipality - Collaboration from community - Increased awareness in the community - Involvement of community in search and destroy - Self satisfaction - Lack of awareness in community - Unaware about potential breeding site - Planning after outbreak - Community negligence - Conflict during search and destroy - Scarcity of water - Social practice - Cultural practice - Shortage of medicine - Shortge of dengue testing kits - Increased case and outbreak - Municipality order - Job obligation - Willing to continue preventive activity - Need of proactive planning - Future plans - Requirement of coordination - Need of self-responsive community - Community empowerment | 1. Knowledge regarding dengue 2. Perceived risk of contracting dengue 3. Personal dengue experience 4. Health impact of dengue 5. Economic impact of dengue 6. Emotional Impact of dengue 7. Home to home visit 8. Awareness campaign 9. Search and destroy campaign 10. Collaboration 11. Increased awareness 12. Community engagement 13. Sense of fulfilment 14. Knowledge gaps in community 15. Reactive planning rather than proactive planning 16. Community reluctance 17. Scarcity of water as challenges 18. Socio-cultural practice 19. Shortage of paracetamol and testing kits 20. Outbreaks as catalysts 21. Municipality order 22. Job role obligations 23. Confidence in Personal Contribution 24. Future Plans 25. Proactive awareness and Prevention 26. Government Coordination and Action 27. Community empowerment and responsibility | 1. Knowledge regarding dengue 2. Perceived risk of contracting dengue 3. Personal dengue experience 4. Health,Economic and Emotional Impact 5. Contribution during dengue outbreak 6. Increased awareness 7. Community engagement 8. Sense of fulfilment 9. Knowledge gaps in community 10. Reactive planning mechanism 11. Community reluctance 12. Scarcity of water as challenges 13. Socio-cultural practice 14. Shortage of paracetamol and testing kits 15. Outbreaks as catalysts 16. Municipality order 17. Job role obligations 18. Confidence in Personal Contribution 19. Future Plans 20. Proactive awareness and Prevention 21. Government Coordination and Action 22. Community empowerment and responsibility | 1. Perceived Susceptibility 2. Perceived Severity 3. Perceived Benefits 4. Perceived Barriers 5. Cues to Action 6. Self-Efficacy |
