# Supplementary material for: A qualitative exploration of community stakeholders perspectives on dengue outbreak management in urban Nepal: navigational insights and challenges
Source: Trop Med Health. 2025 May 30;53:77. doi: 10.1186/s41182-025-00758-w (PMC12123987; doi:10.1186/s41182-025-00758-w)
Supplement: Supplementary file 2 — Additional file 2. Table S2: Socio-demographic information of the research participants [file 41182_2025_758_MOESM2_ESM.docx]

**S2 Table: Socio-demographic information of the research participants.**

| **Code** | **Age** | **Sex** | **Education status** | **Employment Details with years (Yrs)of experience** |
| --- | --- | --- | --- | --- |
| KII 1 | 51-55 | Female | Higher secondary education | THP for 3-4 yrs and FCHV since last 3 yrs |
| KII2 | 41-45 | Female | Secondary Education | FCHVs since 15 yrs |
| KII 3 | 36-40 | Female | Higher secondary education | FCHVs since 10 yrs |
| KII 4 | 41-45 | Female | Secondary Education | FCHVs since 13 yrs |
| KII 5 | 51-55 | Female | Secondary Education | FCHVs since 5-7 yrs |
| KII 6 | 56-60 | Female | Secondary Education | FCHVs since 18-20 yrs |
| KII 7 | 41-45 | Female | Secondary Education | THP since 5-7 yrs |
| KII 8 | 41-45 | Female | Secondary Education | THP since 10 yrs |
| KII 9 | 56-60 | Female | Secondary Education | THP for 2-3 yrs and FCHVs since last 13yrs |
| KII 10 | 46-50 | Female | Higher secondary education | FCHVs since 18-20 yrs |
| KII 11 | 56-60 | Female | Secondary Education | FCHVs since 18-20 yrs |
| KII 12 | 45-50 | Female | Higher Education | FCHVs since 18-20 yrs |
| KII 13 | 51-55 | Female | Secondary Education | FCHVs Since 16 yrs |
| KII 14 | 50-55 | Female | Secondary Education | FCHVs since 18-20 yrs |
| KII 15 | 50-55 | Female | Higher secondary education | FCHVs since 26 yrs |
| KII 16 | 46-50 | Male | Higher secondary education | Ward representatives (ward chairperson) |
| KII 17 | 46-50 | Male | Secondary Education | Ward representatives (ward member) |
| KII 18 | 60-65 | Male | Higher Education | Ward representatives (ward chairperson) |
| KII 19 | 50-55 | Male | Secondary Education | Ward representatives (ward member) |
| KII 20 | 36-40 | Male | Higher secondary education | Ward representatives (ward member) |
| KII: Key informant interview; THP: Tole Health Promoter ; FCHVS: Female Community Health Volunteer | | | | |
